# A Rare Case of Pituitary Apoplexy Secondary to Dengue Fever-induced Thrombocytopenia

**DOI:** 10.7759/cureus.5323

**Published:** 2019-08-05

**Authors:** Mathew Thomas, Alex Robert, Pavan Rajole, Priya Robert

**Affiliations:** 1 Department of Breast Medical Oncology, Cleveland Clinic, Cleveland, USA; 2 Internal Medicine, Church of South India Holdsworth Memorial Hospital, Mysore, IND; 3 Internal Medicine, Government Medical College, Kottayam, IND

**Keywords:** pituitary apoplexy, dengue, thrombocytopenia

## Abstract

Pituitary apoplexy (PA) is an endocrine emergency resulting from hemorrhage or infarction within a pituitary tumor or non-tumorous pituitary gland. The most important predisposing factors for PA are cerebral angiographic procedures, systemic hypertension, surgeries, head injury, coagulopathies, and drugs. Thrombocytopenia is a risk factor for PA. Dengue fever causes thrombocytopenia and there are reported cases of dengue hemorrhagic fever predisposing to PA. But there are no reported cases of dengue fever per se predisposing to PA, and we report such a case in an 85-year-old elderly male who presented with features suggestive of a hypertensive emergency and, on evaluation, was found to have a pituitary incidentaloma and dengue fever. During the hospital course, he developed acute III^rd ^nerve palsy and, when evaluated, was found to have PA. He responded well to medical management with steroids and thyroxine. Prompt initiation of treatment is of utmost importance in pituitary apoplexy, as it can result in adverse events, including loss of vision and even death from hemodynamic compromise.

## Introduction

Pituitary apoplexy (PA) is an acute clinical syndrome characterized by sudden-onset headache, vomiting, visual disturbances, altered sensorium, and ophthalmoplegia, second­ary to hemorrhage or infarction within a pituitary tumor or non-tumorous pituitary gland [1]. PA may occur spontaneously or as a result of multiple risk factors [2]. Dengue fever causes thrombocytopenia, which, in turn, can precipitate PA. A review of the literature showed five reported cases of dengue hemorrhagic fever predisposing to PA [1-5]. However there are no reported cases of dengue fever per se predisposing to PA, and we report such a case of PA in the setting of dengue fever-induced thrombocytopenia.

## Case presentation

An 85-year-old elderly male presented with a low-grade fever for three days, generalized headache for two days, and one day of giddiness. His headache was gradual in onset and of a dull, aching type. There was no history of vomiting, altered sensorium, seizures, head trauma, or weakness. Past medical history was significant for presbycusis and hypertension (on 5 mg PO amlodipine). He denied any history of smoking, excessive alcohol use, or substance abuse. On examination, he was alert and oriented, with a pulse rate of 68/min, blood pressure (BP) of 210/120 mmHg, and respiratory rate of 18/min. The physical examination was unremarkable except for bilateral sensorineural hearing loss.

Investigations and treatment

Labs at presentation (Table 1) were significant for thrombocytopenia.

**Table 1:** Labs at presentation MCV: mean corpuscular volume; TSH: thyroid stimulating hormone

Variable	Reference values	Measurement
Hemoglobin (g/dL)	13.5-17.5	14
Total leucocyte count (TLC) (/mm3)	4,500-11,000	4400
Platelet count (/mm3)	150,000 - 400,000	9000
MCV (μm3)	80-100	85
Sodium (mEq/L)	136-145	141
Potassium (mEq/L)	3.5-5.0	4.3
Blood urea nitrogen (mmol/dL)	8–24	26
Creatinine (mg/dL)	0.6-1.2	1.2
TSH (µU/mL)	0.5-5	0.63

**Figure 1:**
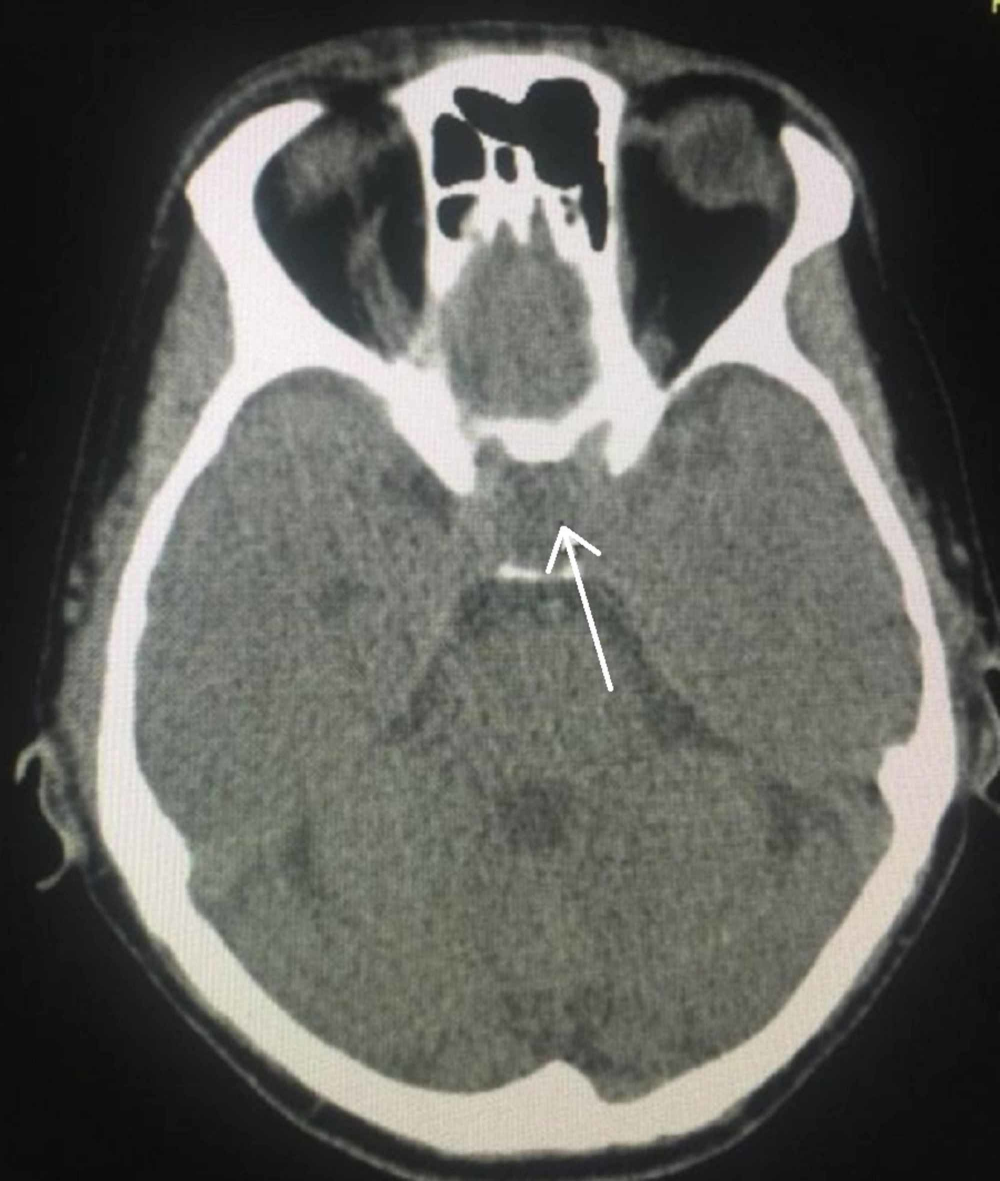
Computed tomography brain showing an iso to hypodense lesion in the sella (arrowhead), causing sellar widening, suggestive of a pituitary lesion, likely a pituitary macroadenoma

His electrocardiogram (ECG) showed sinus rhythm with first-degree heart block. His CT brain showed a sellar lesion favoring a pituitary macroadenoma (25 x 24 mm) (Figure 1). In view of his fever and thrombocytopenia, he was evaluated for dengue fever and was found to have dengue immunoglobulin M (IgM) positive, and hence his platelet count was monitored daily (Table 2). Thus, his differential diagnoses were a hypertensive emergency, severe thrombocytopenia, most probably post viral, and pituitary incidentaloma.

**Table 2:** Daily platelet recordings

Variable	Reference value	Measurement			
		Day 1	Day 2	Day 3	Day 4
Platelet count (/mm3)	150,000- 400,000	9000	48,000	29,000	10,000

**Figure 2:**
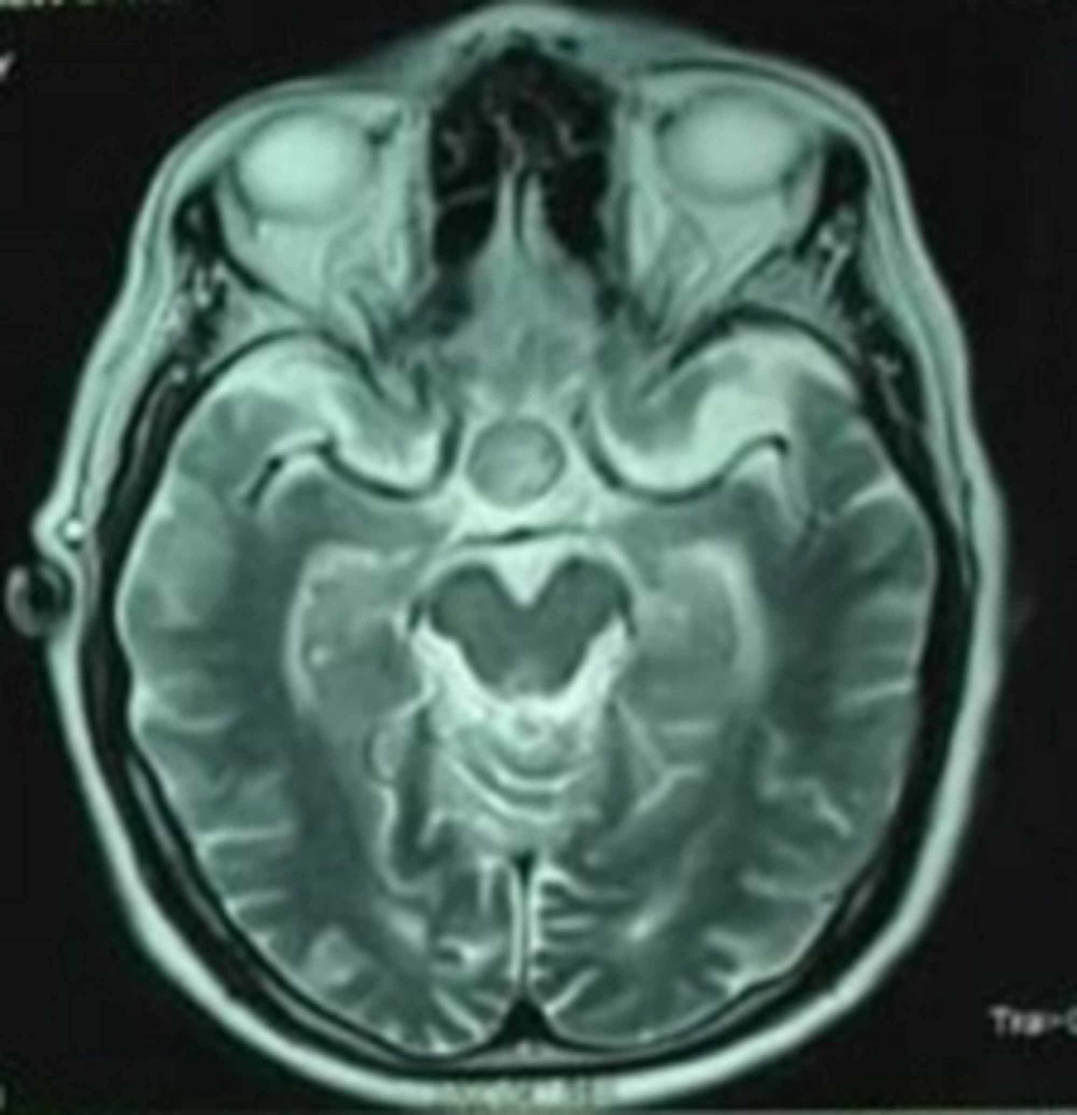
MRI brain showing pituitary macroadenoma MRI: magnetic resonance imaging

**Figure 3:**
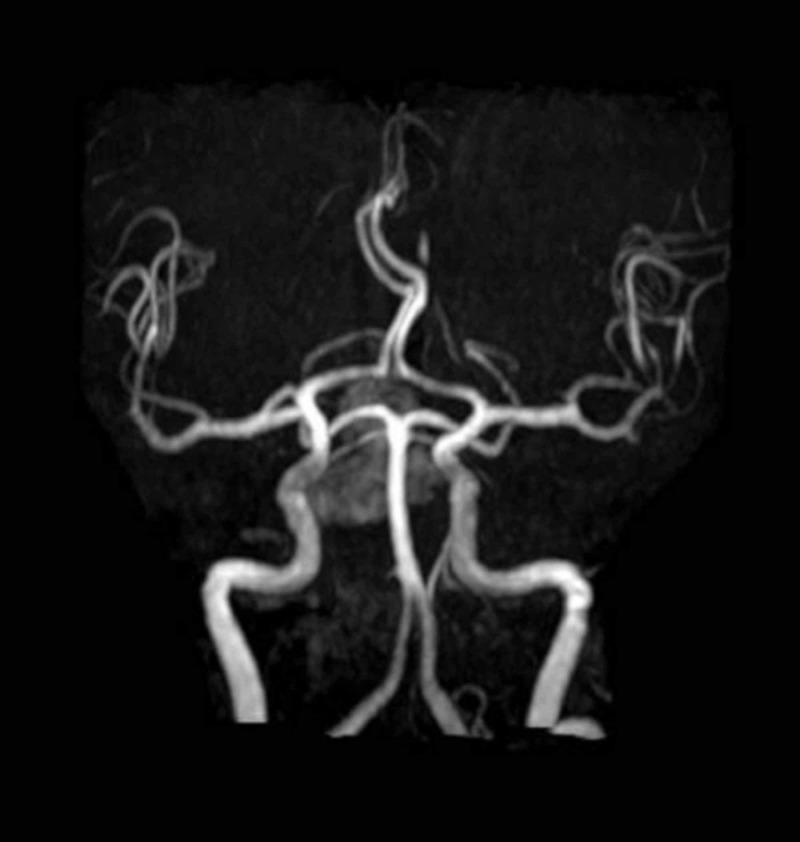
Normal MR angiogram MR: magnetic resonance

He showed clinical improvement with symptomatic management. But unfortunately on Day 3 of his hospital stay, he became restless, his headache reappeared, and it was associated with neck stiffness. A neurological examination revealed the presence of ptosis and a divergent squint in the right eye; pupils were mid-dilated and sluggish in reaction, the left eye was normal, and there were no signs of meningeal irritation. In view of his incidentaloma, serum prolactin assay (Table 3) and a magnetic resonance imaging (MRI) plus MR angiogram of the brain was done, which revealed a pituitary macroadenoma with normal MR angiogram findings (Figures 2-3). He was treated with mannitol and responded well.

**Table 3:** Pituitary hormonal assay f T4: free T4; TSH: thyroid-stimulating hormone

Variable	Reference value	Measurement	
		Day 1	Day 4
Cortisol (µg/dL)	6.7-22.6	6.88	
TSH (µU/mL)	0.5-5	0.63	0.22
f T4 (µg/dL)	5-12	0.75	0.67
Prolactin (ng/mL)	< 20	1.67	

**Figure 4:**
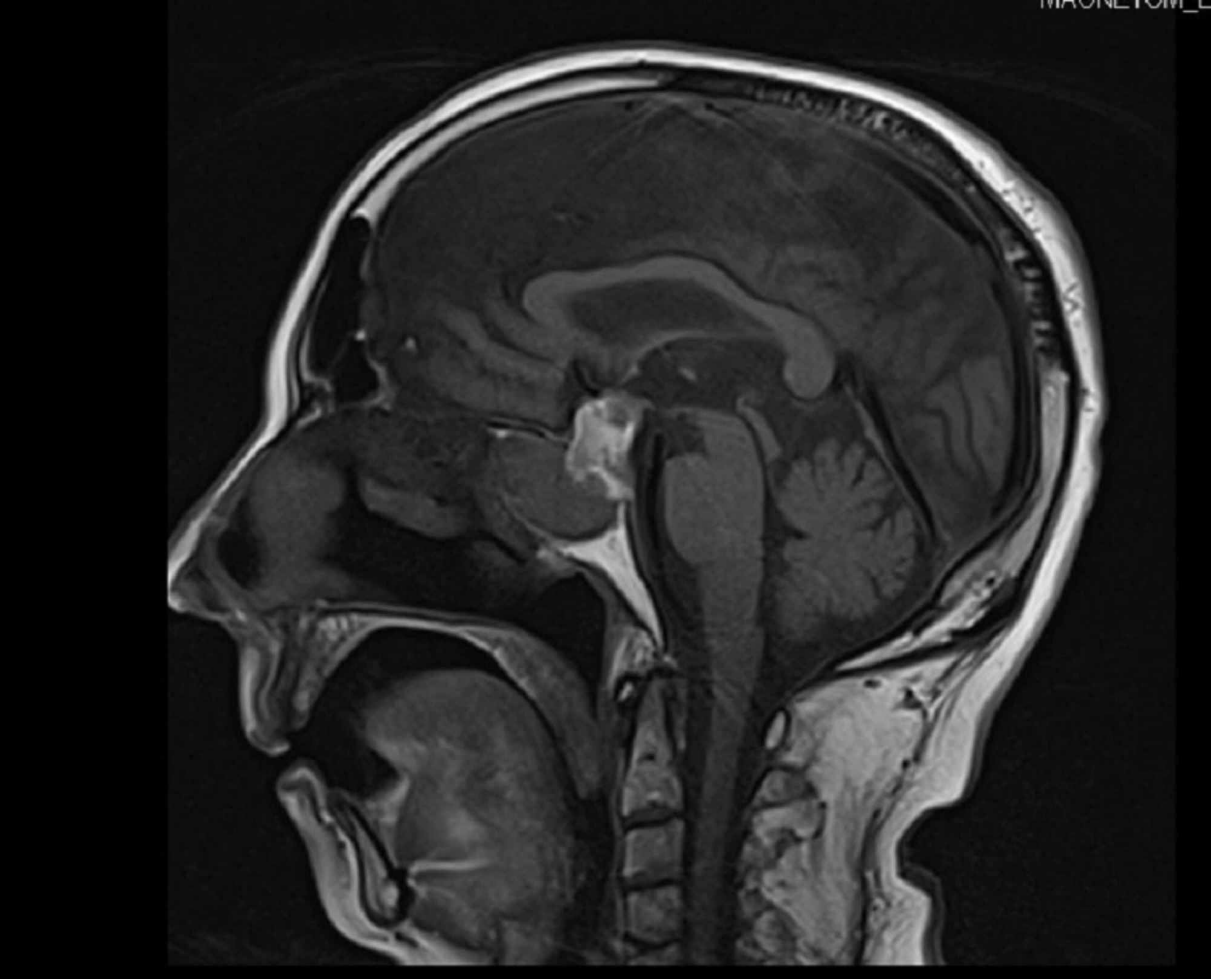
MRI brain showing pituitary macroadenoma with hemorrhagic areas. MRI: magnetic resonance imaging

But on Day 4, his sensorium worsened, BP dropped to 90/60 mmHg, serum sodium was 128 mEq/L, and platelet count dropped to 10,000. Due to the acute onset development of hypotension from the initial presentation of hypertension, associated with hyponatremia in the background of a pituitary incidentaloma, secondary adrenal insufficiency was suspected. Hence, mannitol was replaced with hydrocortisone (after serum cortisol analysis), and four units of platelet transfusions were given. With steroids, he showed significant clinical and hemodynamic improvement, but his ptosis persisted. Repeat brain MRI was done and showed a pituitary macroadenoma (24 x 22mm) with hemorrhagic areas, consistent with the diagnosis of pituitary apoplexy (Figure 4). His pituitary hormonal assay showed decreased levels of thyroxine and prolactin and a lower limit of normal of cortisol (Table 3).

He responded well to steroids, and hence there was no indication for surgical intervention. He was also supplemented with thyroxine 50 µg PO daily. He was discharged with PO medications of hydrocortisone (20 mg in the morning and 10 mg in the evening) and thyroxine 50 µg daily. A follow-up visit at two weeks showed a significant improvement of ptosis and full recovery in two months.

## Discussion

We report a case of pituitary apoplexy in an elderly male from dengue fever-induced thrombocytopenia. The incidence of pituitary apoplexy in pituitary tumors is about 2% - 12% [6].The important risk factors for PA are cerebral angiographic procedures, systemic hypertension, surgeries (cardiac and orthopedic), head injury, coagulopathies, and drugs (GnRH analogs, dopamine receptor analogs, etc.) [7]. The pathophysiological process involved in PA are: (a) reduced blood flow resulting in infarction, (b) acute increase in blood flow, (c) stimulation of the pituitary gland from stress tests, and (d) coagulopathies from thrombocytopenia or anticoagulation [8]. Our patient had thrombocytopenia from dengue fever, which precipitated apoplexy.

The clinical features of PA include headache, signs of meningeal irritation, visual disturbances (due to sudden hemorrhage-related changes, or inflammation of the optic nerve from the bleed), and features of oculomotor (III^rd^) nerve palsy [7]. Acute endocrine deficiency also ensues, which makes PA an endocrine emergency. The most common hormonal deficiency is corticotrophin deficiency, occurring in up to 80% of cases, resulting in severe hemodynamic instability and hyponatremia [7]. PA can also result in other hormonal deficits like growth hormone (GH), thyrotropic, and gonadotropic deficiency [7].

CT brain is used for the initial evaluation; MRI is the investigation of choice. PA can be managed both medically and surgically, but the most appropriate approach in the acute phase is controversial [7]. Medical management is aimed at hemodynamic stabilization, correction of electrolyte imbalance, and empiric parenteral steroids (preferably after a blood draw for cortisol assessment), as secondary adrenal insufficiency can occur from acute corticotrophin deficiency [7-8]. Surgery is considered if the patient has a progressive loss of vision and deterioration of consciousness [6]. Transsphenoidal surgery is the recommended surgical approach [7]. Our patient was managed medically with steroids and thyroxine and showed drastic improvement, and hence surgery was not indicated.

Pituitary apoplexy is thus an endocrine emergency and if corticosteroids are not started immediately, death may follow as a result of adrenal failure or other neurological complications.

## Conclusions

Dengue fever is a known risk factor for thrombocytopenia, which, in turn, can precipitate pituitary apoplexy. When a patient with thrombocytopenia in the background of a pituitary adenoma (known case of adenoma or incidentaloma) develops vomiting, headache, or meningeal irritation, with features of acute III^rd^ nerve palsy, pituitary apoplexy should be kept in mind and intervened immediately, as it is an endocrine emergency that can result in adverse clinical outcomes.
